# Antimicrobial Susceptibility of Toxin-Producing *Corynebacterium diphtheriae* and *C. ulcerans* in Belgium

**DOI:** 10.3390/antibiotics14020160

**Published:** 2025-02-06

**Authors:** Zan Janssen, Helena Martini, Robin Vanstokstraeten, Kristof Vandoorslaer, Ingrid Wybo, Eveline Van Honacker, Denis Piérard

**Affiliations:** National Reference Center for Toxigenic Corynebacteria, Department of Microbiology and Infection Control, Universitair Ziekenhuis Brussel (UZ Brussel), Vrije Universiteit Brussel (VUB), 1090 Brussels, Belgium; zanjanssen@gmail.com (Z.J.); helena.martini@uzbrussel.be (H.M.); robin.vanstokstraeten@hotmail.com (R.V.); ingrid.wybo@uzbrussel.be (I.W.); eveline.vanhonacker@uzbrussel.be (E.V.H.)

**Keywords:** diphtheria, *Corynebacterium diphtheriae*, *Corynebacterium ulcerans*, antimicrobial susceptibility, antimicrobial resistance determinants, whole genome sequence

## Abstract

**Background/Objectives**: Despite a significant reduction in diphtheria incidence and mortality due to vaccination, antitoxin therapy and antibiotic treatments, a concerning resurgence is occurring in Europe. Resistance to penicillins and macrolides is emerging, resulting in a growing challenge for diphtheria management. This retrospective study aims to evaluate and compare antibiotic susceptibilities of both toxigenic *Corynebacterium diphtheriae* and *C. ulcerans*. **Methods**: Susceptibilities were assessed using broth microdilution—the gold standard—disk diffusion and the gradient method, and analyzed on the basis of the EUCAST breakpoint tables for the interpretation of MICs and zone diameters. Antimicrobial resistance genes and mutations were detected by analyzing whole-genome sequences (WGS). **Results**: A small number of *C. diphtheriae* isolates were resistant to the first-choice antimicrobial classes, penicillins and macrolides, while higher resistance rates were observed for ciprofloxacin (29%), tetracycline (38%) and trimethoprim-sulfamethoxazole (SXT, 85%). A good correlation was found with resistance genes and mutations detected by WGS. *C. ulcerans* isolates were susceptible to all tested antibiotics, except clindamycin, to which this species is naturally resistant, and a few ciprofloxacin resistances not confirmed by WGS. Diffusion techniques were found to be acceptable alternatives, but false susceptible results were detected for ciprofloxacin and tetracycline by disk diffusion and ciprofloxacin and SXT by gradient diffusion. **Conclusions**: Penicillins and macrolides remain the first-choice antibiotics for the treatment of diphtheria. However, antimicrobial susceptibility testing is needed for all toxigenic *C. diphtheriae* and *C. ulcerans* isolates, as resistance is emerging. Antimicrobial susceptibility testing should not be limited to penicillins and macrolides, but be extended to other antibiotics. When WGS is performed for epidemiological purposes, resistance genes and mutations should be looked for.

## 1. Introduction

Toxigenic *Corynebacterium diphtheriae*, *Corynebacterium ulcerans* and (rarely) *Corynebacterium pseudotuberculosis*, *Corynbacterium belfantii*, *Corynebacterium rouxii* and *Corynebacterium silvaticum* are Gram-positive bacilli that potentially produce the diphtheria toxin [1]. These species are the agents of diphtheria, which is a potentially fatal upper respiratory disease or a cutaneous disease [2,3]. These organisms can infect the pharynx, tonsils and nasal tract, resulting in tonsillitis, laryngitis or pharyngitis with pseudomembrane in the nasopharynx and laryngeal vestibule. The diphtheria toxin is an exotoxin, responsible for the potentially fatal systemic complications of this disease, encoded by the tox gene and carried by lysogenized corynebacteriophages within the bacterial chromosome. The principal modes of transmission between humans are airborne droplets or direct contact with cutaneous lesions. Humans are the main reservoir of *C. diphtheriae*, although it has also been isolated from a few domestic animals. *C. ulcerans* is a zoonotic pathogen that colonizes domestic and wild animals and is occasionally transmitted to humans.

Of note, non-lysogenized strains of these species harbored by healthy carriers do not produce the toxin and are not a cause of diphtheria [2,3]. Despite the significant reduction in diphtheria incidence and mortality due to the effective use of vaccines, antitoxin therapy and antibiotic treatments, a concerning resurgence is occurring in Europe. In 2022, 363 cases of diphtheria were reported in migrant centers of six European countries including the UK, mostly among people with a recent migration history and/or close contact with migrant populations and/or a recent date of entry into the reporting country [4]. From 2016 to 2021, the number of *C. diphtheriae* cases reported by European Union/European Economic Area (EU/EEA) countries (excluding the UK) in the European Centre for Disease Prevention and Control (ECDC) Surveillance Atlas of Infectious Diseases ranged from 13 to 30 cases, followed by 318 cases in 2022 and 135 in 2023, but only 29 in 2024 up to 31 December 2024; during the same period, the number of reported cases of *C. ulcerans* raised from 19 to 42 in 2023 but decreased to 21 in 2024 [5]. Belgium confirmed 25 cases of toxigenic *C. diphtheriae* infection among asylum seekers in 2022 and 7 cases in 2023 [6].

The eradication of diphtheria is primarily achieved through the Diphtheria, Pertussis and Tetanus (DPT) immunization program. This vaccination regimen is widely implemented in Europe and is being progressively introduced in developing countries. Vaccination schedules typically include three doses administered between 4 weeks and 3 months of age and boosters at 15 months and at 6 years. Additionally, booster doses every ten years are officially recommended to maintain immunity [7]. However, a recent European seroprevalence study in middle-aged adults showed a high proportion of sera with unprotected levels for diphtheria, raising concerns about possible spread of the disease [8].

The treatment of diphtheria consists of early administration of diphtheria anti-toxin (DAT), which neutralizes the unbound toxin when administered within the first 48 h of symptoms [2]. Eradication of the organism by antibiotic therapy prevents further toxin production as well as transmission. Penicillin and macrolides are the primary antibiotics used to treat infections caused by toxigenic *C. diphtheriae* and *C. ulcerans* [2]. However, antibiotic resistance is emerging and results in a growing challenge in the effective management of diphtheria cases [9,10,11,12,13,14,15]. The current patterns of migration and associated health risks further complicate this issue, potentially leading to an increase in antibiotic-resistant diphtheria infections [4,6].

Until 2023, there had been no consensual antimicrobial susceptibility testing (AST) methods and interpretations for the agents of diphtheria, as the Clinical and Laboratory Standards Institute (CLSI) and the European Committee on Antimicrobial Susceptibility Testing (EUCAST) changed their recommendations several times [16]. In 2023, EUCAST introduced specific breakpoints for *C. diphtheriae* and *C. ulcerans* in the “Breakpoint tables for interpretation of MICs and zone diameters. Version 13.0, 2023”, which remained unchanged in versions 14.0, 2024, and 15.0, 2025 [16,17].

This retrospective study aims to evaluate and compare the antibiotic susceptibilities of both *C. diphtheriae* and *C. ulcerans* isolated in Belgium from 2010 to 2023. Susceptibilities were assessed using broth microdilution (BMD), which is considered the golden standard, disk diffusion and the gradient method. In addition, antimicrobial resistance (AMR) determinants, i.e., resistance genes and point mutations of *gyrA*, were detected by analyzing whole-genome sequences (WGS).

## 2. Results

### 2.1. Broth Microdilution (BMD)

**Figure 1:**
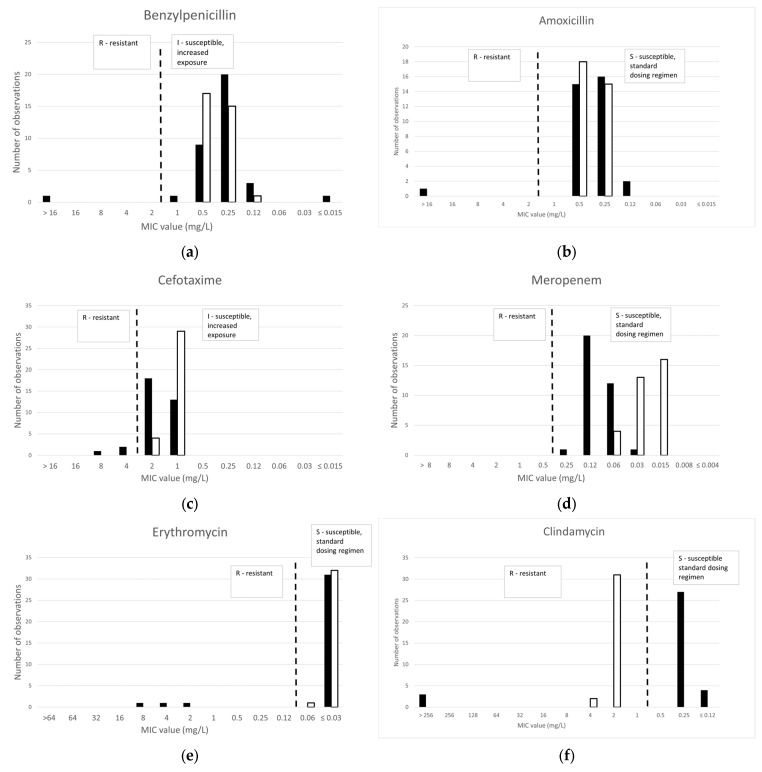

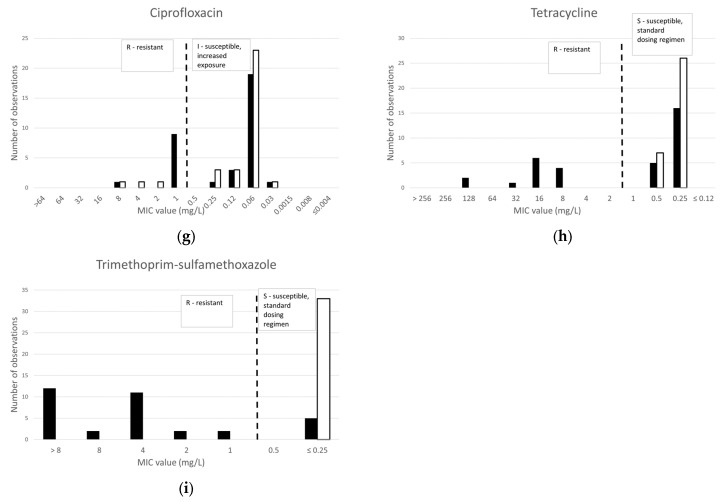
MIC distributions per antibiotic obtained by broth microdilution (BMD) for 34 isolates of *C. diphtheriae* (black bars) and 33 isolates of *C. ulcerans* (white bars). EUCAST MIC breakpoints (EUCAST Breakpoint Tables v. 14.0 [16,17]) are shown as dotted lines (R/I for benzylpenicillin, cefotaxime, ciprofloxacin; R/S for amoxicillin, meropenem, erythromycin, clindamycin, tetracycline, trimethoprim-sulfamethoxazole). MICs for trimethoprim/sulfamethoxazole are expressed as trimethoprim concentrations.

Figure 1 shows the distribution of minimal inhibitory concentration (MIC) of the human toxicogenic clinical isolates of *Corynebacterium diphtheriae* (34 isolates) and *Corynebacterium ulcerans* (33 isolates) collected during the years 2010 to 2023. Non-toxicogenic isolates and isolates from animals were not included.

AMR determinants obtained by the analysis of WGS are presented in Table 1 and will be discussed together with the results of the antibiotics susceptibility testing using the three studied techniques in the following sections.

### 2.1.1. β-Lactams

Only one *C. diphtheriae* strain was resistant to benzylpenicillin, amoxicillin and cefotaxime, and this resistance was mediated by the presence of a modified penicillin-binding protein, encoded by the *pbp2m* gene. The presence of a β-lactamase gene, *bla-OXA2*, did not lead to the resistance or elevation of MIC values. The range of MIC values to cefotaxime was higher as compared to the other tested β-lactams, and two more *C. diphtheriae* were classified as resistant in spite of the absence of detected AMR. All *C. diphtheriae* isolates were susceptible to meropenem.

No resistance to β-lactam antibiotics was observed in any of the 33 *C. ulcerans* isolates, and no AMR was identified.

#### 2.1.2. Macrolides and Lincosamides

Three *C. diphtheriae* isolates, including the β-lactam resistant isolate, were resistant to erythromycin and clindamycin and carried the *ermX* gene. All *C. ulcerans* isolates were susceptible to erythromycin and resistant to clindamycin, but no AMR was detected.

#### 2.1.3. Other Antibiotics

Three antibiotics not usually recommended for the treatment of diphtheria were tested as alternatives for the treatment of infections with multi-resistant strains. Ciprofloxacin, tetracycline and trimethoprim-sulfamethoxazole (SXT) showed resistance rates of, respectively, 29%, 38% and 85% for *C. diphtheriae* and 9%, 0% and 0% for *C. ulcerans*.

Ciprofloxacin, as a representative of the fluoroquinolone family, was found to be susceptible in 24 of the 34 *C. diphtheriae isolates* (71%), but 10 isolates were resistant. There was a good correlation with the presence of AMR determinants, which were detected in all these ciprofloxacin resistant strains, the S89F mutation in the *gyrA* gene in two strains, *qacE*Δ in two strains and both in six strains. However, *qacE*Δ and *qacL* genes were also detected in one susceptible isolate, with a low MIC of 0.06 mg/L. All but three isolates of *C. ulcerans* were susceptible to ciprofloxacin (91%), but no AMR determinants were identified in the three resistant isolates by WGS analysis.

Tetracycline, as representative of this class of antibiotics, was found to be susceptible in 21 of the 34 *C. diphtheriae* isolates (62%) and all *C. ulcerans* isolates. Tetracyline resistance genes, *tet*, were found in all 13 resistant strains.

As many as 29 of the *C. diphtheriae* isolates were found to be resistant to SXT (only 15% susceptible), while all *C. ulcerans* isolates were susceptible. The resistance gene *sul1*, with or without *dfrA*1 or *dfrA*16 resistance genes, was detected in 27 of these isolates, but also in one susceptible strain with an MIC of ≤0.25 mg/L.

### 2.2. Disk Diffusion Susceptibility Testing

**Figure 2:**
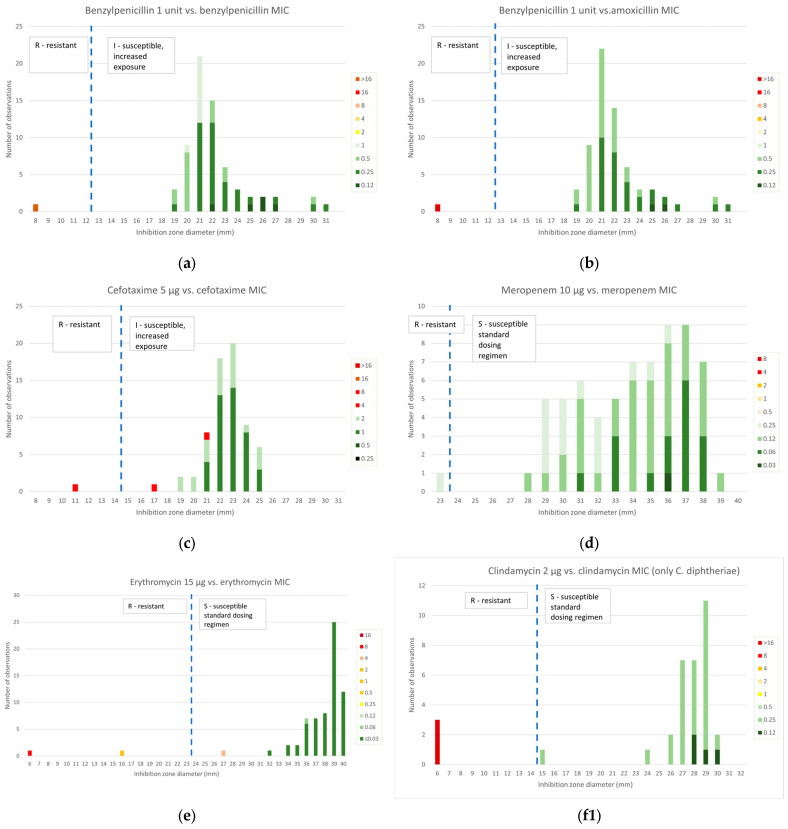

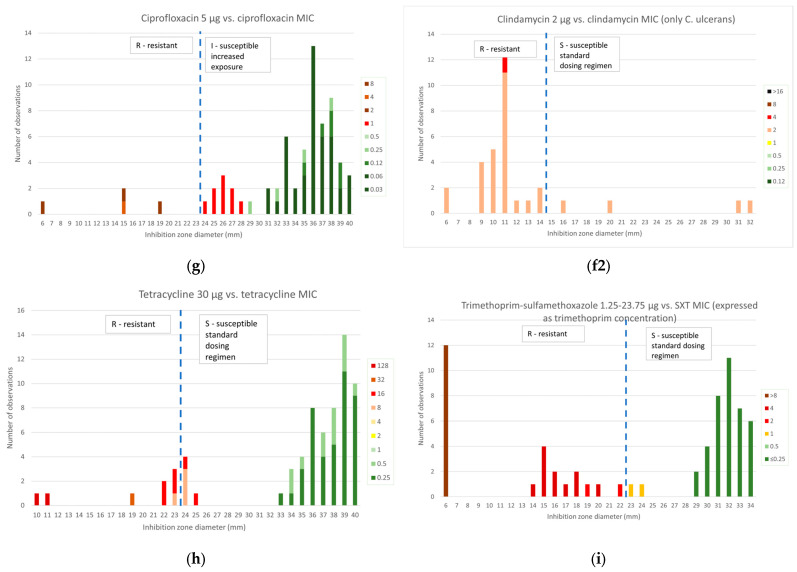
Distribution of disk diffusion inhibition zone diameters as compared with broth microdilution MIC values as colored bars (red–yellow color range for resistant MIC values, green color range for susceptible MIC values) for 34 isolates of *C. diphtheriae* and 33 isolates of *C. ulcerans*. EUCAST zone diameter breakpoints (EUCAST Breakpoint Tables v. 14.0 [16,17]) are shown as dotted lines (R/I for benzylpenicillin, cefotaxime, ciprofloxacin; R/S for amoxicillin, meropenem, erythromycin, clindamycin, tetracycline, trimethoprim-sulfamethoxazole). MICs for trimethoprim/sulfamethoxazole are expressed as trimethoprim concentrations. Clindamycin results for *C. diphtheriae* and *C. ulcerans* are presented separately on graphs (**f1**) and (**f2**), respectively.

The inhibition zone diameters obtained by disk diffusion susceptibility testing for the studied antibiotics were compared to MIC values obtained by BMD, the reference technique, and are presented in Figure 2.

### 2.2.1. β-Lactams

Amoxicillin MIC values were compared to benzylpenicillin inhibition diameters because no zone diameter breakpoints are given for this antibiotic, but EUCAST mentions that isolates “susceptible, increased exposure” (I) to benzylpenicillin can be reported as susceptible to amoxicillin while isolates resistant to benzylpenicillin should be tested for susceptibility to amoxicillin or reported resistant.

A very high concordance was found: 100% for the benzylpenicillin disk as compared to both benzylpenicillin and amoxicillin MIC values, 97% for cefotaxime and 99% for meropenem. Two *C. diphtheriae* isolates categorized as resistant to cefotaxime by BMD with an MIC of 4 mg/L were found to be susceptible by disk diffusion. Of note, no AMR determinants were detected in these isolates. One *C. diphtheriae* isolate was classified as resistant to meropenem with a narrow inhibition diameter of 23 mm.

#### 2.2.2. Macrolides and Lincosamides

**Figure 3:**
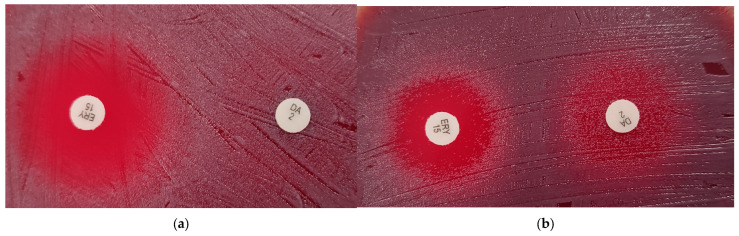
Inhibition zone of two *ermX*-positive strains around erythromycin (ERY15) and clindamycin (DA2) disks. (**a**) Isolate Dift070, (**b**) isolate Dift238.

As can be seen in Figure 2e, the interpretation for erythromycin was concordant with MIC values in 66 of the 67 strains (99%). The inhibition zone of one of the three *ermX*-positive isolates of *C. diphtheriae*, Dift070, was 27 mm; susceptible, despite a MIC of 4 mg/L. This isolate grew in contact with the clindamycin disk and the inhibition zone of erythromycin was unclear, as can be seen in Figure 3a. By contrast, a second *ermX*-positive isolate, Dift238, presented numerous colonies within an inhibition zone around clindamycin, which was interpreted as growth to the disk and a zone of inhibition around erythromycin with numerous colonies to a diameter of 16 mm, interpreted as resistant.

All *C. ulcerans* were susceptible to erythromycin, as expected from the MIC values. As both species behave very differently to clindamycin, two graphs are presented for clindamycin: the correlation was perfect for *C. diphtheriae* (Figure 2(f1)), as no inhibition zone was observed for the three resistant isolates. For *C. ulcerans* (Figure 2(f2)), we observe that although there was a very narrow range of MICs of 2 or 4 mg/L, the diameter spread was very large (6 to 32 mm), and five isolates had inhibition diameters classifying them as susceptible. The concordance was only 85% (27/33).

#### 2.2.3. Other Antibiotics

Of the ten isolates positive for fluoroquinolone AMR determinants, nine presented a MIC for ciprofloxacin of 1 mg/L, the first resistant value, and clustered together as a separate group with diameters of 24 to 28 mm, with all but one being erroneously categorized as susceptible by disk diffusion; one strain with an MIC of 8 mg/L was clearly resistant with growth in contact with the disk. The correlation was only 74% (9 false susceptible/34 *C. diphtheriae* isolates).

A 100% correlation was observed for *C. ulcerans*. The three isolates with high MIC values also had small diameters and were classified as resistant by both techniques despite the absence of detection of AMR determinants.

The correlation of BMD and disk diffusion testing was only 95% (62/67) for tetracycline, and this also presented a population with zone diameters around the breakpoint of 19 to 25 mm, resulting in 5 *C. diphtheriae* isolates with a *tet* gene reported as susceptible by disk diffusion.

Finally, the correlation was very good for SXT as well (61/67, 91%), although two *C. diphtheriae* strains without AMR determinants were reported as resistant by BMD (MIC: 1 mg/L), appearing to be susceptible by disk diffusion.

### 2.3. Gradient Diffusion Susceptibility Testing

#### 2.3.1. β-Lactams

**Figure 4:**
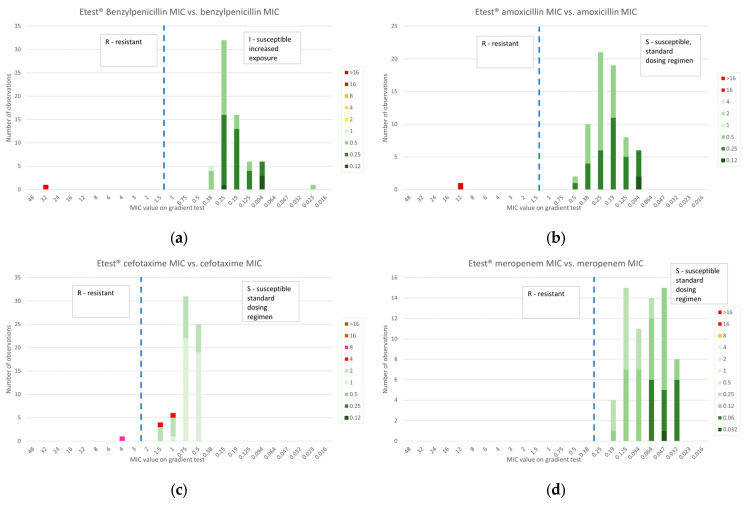

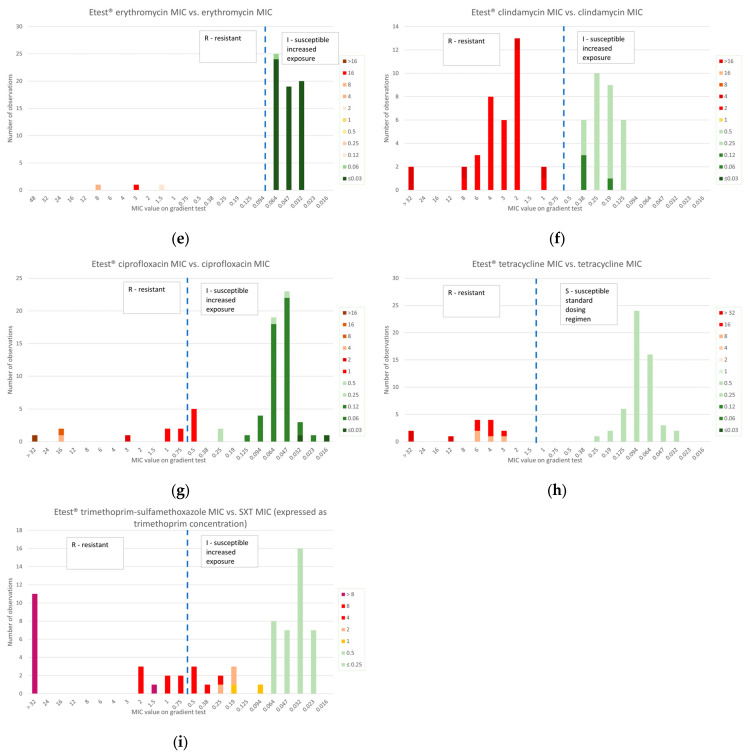
MIC distribution of β-lactam antibiotics obtained with gradient testing as compared with BMD MIC values as colored bars (red–yellow color range for resistant MIC values, green color range for susceptible MIC values) for 34 isolates of *C. diphtheriae* and 33 isolates of *C. ulcerans*. EUCAST zone diameter breakpoints (EUCAST Breakpoint Table 14.0 [16,17]) are shown as dotted lines (R/I for benzylpenicillin, meropenem; R/S for amoxicillin, cefotaxime).

In Figure 4, β-lactam MIC values obtained by gradient diffusion and BMD were compared. A perfect categorical correlation was observed for penicillin, amoxycillin and meropenem. Two isolates with resistant MIC values of 4 mg/L by BMD despite the absence of AMR determinants were classified as susceptible by gradient diffusion.

##### 2.3.2. Macrolides and Lincosamides

In Figure 4, erythromycin and clindamycin MIC values obtained by gradient diffusion were compared to those obtained by BMD. A perfect correlation of categorical classification was observed.

##### 2.3.3. Other Antibiotics

In line with the disk diffusion, the nine *C. diphtheriae* strains with a low level of resistance to ciprofloxacin, confirmed by the presence of AMR determinants, clustered in values around the breakpoint, but four of them showed a MIC value of 0.75 or 1 mg/L, classifying them adequately as resistant. The other five were classified as susceptible. The correlation, including the *C. ulcerans* isolates, was 93% (62/67).

The distribution of tetracycline MIC values was clear-cut, with the isolates with low-level resistance reported as resistant. The correlation was 100%.

Finally, for SXT, 29 *C. diphtheriae* resistant isolates were spread over values ranging from 0.094 to 2 mg/L, resulting in 10 false susceptible isolates. The concordance in terms of categorical classification was only 85% (57/67).

### 2.4. Whole-Genome Sequencing (WGS)

As mentioned above, the presence of AMR determinants was reported together with the antimicrobial susceptibility results. In addition, WGS data were analyzed for multi-locus sequence typing (MLST) of the strains.

The 34 *C. diphtheriae* isolates belonged to 10 individual sequence types (ST). Two STs were predominant and correlated with antimicrobial resistance: twelve strains of ST574 were all resistant to SXT only. Ten strains belonged to ST 377 and were all resistant to ciprofloxacin, tetracycline and SXT, and two of them were also resistant to erythromycin and clindamycin. In addition, three isolates of ST384 were SXT-resistant. Of note, the multi-resistant strain (combined resistance to β-lactams, except meropenem, erythromycin and clindamycin) belonged to ST510. The other types were ST100 (three strains) and ST67, ST217, ST698, ST856 and ST62 (one strain each).

The 33 *C. ulcerans* isolates belonged to four STs, with 15 ST331, 13 ST 332 (including the 3 isolates resistant to ciprofloxacin), 4 ST 328 and 2 ST325.

### 2.5. Comparison of Resistance Prevalence with Other European Studies

The resistance percentages were compared with two recent European studies [10,16], as shown in Table 2 and Table 3. Resistance proportions were found to be similar for most antibiotics, but the resistance of *C. diphtheriae* to ciprofloxacin and trimethoprim-sulfamethoxazole was considerably higher in the present study.

## 3. Discussion

While equine antitoxin administration remains the mainstay of the treatment of respiratory diphtheria, by neutralizing the circulating diphtheria toxin responsible for systemic disease, complications and mortality, antibiotic treatment is of utmost importance to eradicate the micro-organism. In respiratory diphtheria, as well as in cutaneous forms of the disease rarely associated with systemic toxicity, it is important to interrupt the transmission of the micro-organism, in addition to contact and droplet isolation procedures [2]. As such, in respiratory diphtheria, false susceptibility reporting has a more adverse impact on infection control issues like propagation of the micro-organisms, resistance to antibiotics and control of outbreaks than on the cure of the individual patient. Antibiotics have more importance in the cure of cutaneous diphtheria, often multi-microbial [2].

Penicillins and macrolides are most frequently included in therapeutic recommendations [2,18,19]. However, in recent years, sporadic cases of *C. diphtheriae* resistant to penicillin, macrolides or both have been reported [12,14,20,21]. More worryingly, in one large outbreak of diphtheria in Nigeria, all isolates were reported to be resistant to penicillin, but the resistance mechanism was not mentioned [22]. On the other hand, *C. ulcerans* is susceptible to almost all antibiotics, except to clindamycin, to which it is naturally resistant [17].

Penicillin resistance is mediated by a supplementary penicillin-binding protein (PBP2m) discovered by Hennart et al. [9]. In addition, three strains possessed the *blaOXA-2* were also reported by Hoefer et al. [4], but were not detected in the large study by Hennart et al. [9]. This enzyme is an oxacillinase, whose gene is found in diverse Gram-negative bacterial species such as *Acinetobacter*, *Shewanella*, *Pseudomonas* and *Burkholderia*, but also in *Corynebacterium amycolatum* [23]. It hydrolyses penicillins, but also carbapenems. In *E. coli*, *blaOXA-2* can be silenced as the gene and its promoter remain intact, with fully wild-type sequences, but not expressed. The mechanism by which this occurs is unknown, but the process is reversible and this can have clinical implications [23]. Disk and gradient diffusion results correlated well with BMD, except for cefotaxime, where two of the three resistant *C. diptheriae* strains were found to be susceptible, but as no AMR determinants were identified in these strains, we question the BMD result. It should also be noted that MIC values are always higher for cefotaxime, questioning its usefulness in diphtheria treatment.

Ribosomal methylation mediated by the *ermX* (erythromycin ribosomal methylase) gene that inactivates not only the macrolides but also the lincosamides explains the cross-reactivity with clindamycin. It was detected in the three *C. diphtheriae* isolates that were resistant with BMD. This resistance is inducible, and clindamycin is a better inducer than erythromycin, while in *Staphylococcus aureus* and *Streptococcus pyogenes*, it is the opposite [24]. Caution is necessary, and clindamycin-resistant *C. diphtheriae* isolates should be examined carefully for erythromycin resistance or systemically reported as resistant. Of note, clindamycin could be an alternative to macrolides when arrhythmogenic potential is a concern [2]. However, this is not the case for *C. ulcerans*, which is known to present elevated MIC values to clindamycin [16]. In our study, all strains were resistant with BMD, and a few were false-susceptible with the disk diffusion technique. We propose using an expert rule and systematically reporting this species as resistant to clindamycin if tested susceptible.

An important observation in this study is that resistance to fluoroquinolones and SXT is still rising, as compared with the results of two recent surveys performed in two neighboring countries, France and Germany [10,16]. In addition, these resistances are low-level and difficult to detect using diffusion techniques. A group of nine strains with low-level resistance to ciprofloxacin, a promising antibiotic for the treatment of diphtheria despite the lack of clinical data, possessing either the gyrA-SF89F mutation, a qacEΔgene or both, were all misclassified as susceptible by disk diffusion, and five of them were misclassified by gradient diffusion. Such a group of strains with an MIC of 1 mg/L does not clearly appear in the study by Berger et al. [16], but all belonged to ST 377, which spread after Berger’s period of collection (1951–2017), while all our isolates, except for four, were collected in the years 2022 and 2023. It should be interesting to define an “area of technical uncertainty (ATU)” of 25 to 29 mm, a warning to laboratory staff to avoid false susceptibility reports by performing alternative testing like BMD to solve the issue, as explained in the document “Area of Technical Uncertainty (ATU) in antimicrobial susceptibility testing” [25].

Similarly, nine strains, all also belonging to ST 377, formed a separate population of low-level resistance to tetracycline, with disk diffusion diameters of 22 to 25 mm, which were false susceptible. An ATU of 25–26 mm would be useful here. However, no misclassification was observed in the gradient diffusion of tetracycline.

Finally, disk diffusion performed well for SXT, with only two false-susceptible *C. diphtheriae* strains, but ten isolates were false-susceptible in gradient diffusion.

Compared to the study performed by Berger et al. [16] to establish the EUCAST breakpoints, our microbroth dilution results showed comparable results for *C. ulcerans*, i.e., very low resistance rates except for clindamycin. However, three isolates of *C. ulcerans* were resistant to ciprofloxacin, but no AMR determinants were identified. For *C. diphtheriae*, we observed higher resistance rates for ciprofloxacin (28.6% versus 5.7%) and SXT (87.5% versus 32.5%), confirmed by the detection of resistance mutations for the first and resistance genes for the second. This higher prevalence of resistance is maybe due to the different period of collection; while Berger et al. [16] included isolates collected in two neighboring countries, France and Germany, over the years 1951–2017, our collection included only 4 *C. diphtheriae* isolated before 2022, and 30 isolates from the years 2022 and 2023, many of them originating from patients with recent migration. Although one sequence type, ST 377, is associated with resistance to ciprofloxacin, tetracycline and SXT, our isolate collection cannot be qualified as monoclonal, since as many as seven ten sequence types (STs) were identified by analysis of WGSs.

Penicillins and macrolides remain the first-choice antibiotics for the treatment of diphtheria. However, antimicrobial susceptibility testing is needed for all toxigenic *C. diphtheriae* and *C. ulcerans* isolates, as resistance is emerging. Disk diffusion techniques provide acceptable accuracy, but ATUs should be introduced for ciprofloxacin and tetracycline. The gradient diffusion, although slightly more performant for ciprofloxacin and tetracycline, is expensive and does not generate a significant added value as compared to disk diffusion.

Two promising antibiotics, linezolid and rifampicin, could not be included in this study due the lack of inclusion in the custom panels used. However, Berger et al. did not find any resistance to these antibiotics. [16].

Although this study was performed on a limited number of isolates, its strengths are the inclusion of recent isolates, highlighting the increasing resistance to ciprofloxacin and SXT of *C. diphtheriae*, and the use of WGS data to detect AMR determinants and better understand discrepant AST results.

Nowadays, most potentially toxigenic corynebacteria are submitted to reference laboratories like ours, which often perform WGS for epidemiological purposes. It should be advisable to systematically look for AMR determinants to confirm susceptibility testing results.

Finally, since the numbers of toxigenic *C. diphtheriae* and *C. ulcerans* are always very limited, we suggest that reference laboratories consider the production in common of a custom MIC panel to share the costs and allow BMD to be systematically performed.

## 4. Materials and Methods

All available toxigenic human isolates collected by the Belgian National Reference Center (NRC) during a period from 2010 to 2023 were included in this study, consisting of *C. diphtheriae* (n = 34) and *C. ulcerans* (n = 33). These strains were identified by MALDI-TOF MS (MALDI Biotyper; Bruker Daltonics, Bremen, Germany). Toxigenicity was evaluated by real-time polymerase chain reaction (qPCR) and modified Elek test. Nontoxigenic isolates were excluded.

BMD and disk diffusion susceptibility testing were performed according to EUCAST recommendations, including QC with *Streptocccus pneumoniae* ATCC 49619 [26,27,28]. For BMD, as the number of strains was limited for economy reasons, we had to use two existing Sensititre^®^ panels produced by Thermo Fisher Scientific (Basingstoke, UK) intended for other micro-organisms, with MH-F broth, and analyze only antibiotics pertinent for this study. Disk diffusion was performed on MH-F agar with BioRad^®^ antimicrobial susceptibility disks (BioRad, Marnes-la-Coquette, France). The gradient test was performed on MH-F agar with Etest^©^ (bioMérieux, Marcy L’Etoile, France) according to the manufacturer’s instructions. Specifically, the isolates were inoculated onto blood agar plates, followed by incubation for 24 h. The Etest^®^ was then applied to the agar surface using applicator forceps. These plates were incubated at 35 °C for 16–20 h. All minimal inhibition concentrations and disk diffusion inhibition zones were interpreted according to EUCAST Clinical Breakpoint Tables v. 14.0 for *Corynebacterium diphtheriae* and *C. ulcerans* [17].

As the EUCAST recommendations for *C. diphtheriae* or *C. ulcerans* do not include categorical concentrations or zones between either S (“susceptible, standard dosing regimen”) and R (“resistant”) or I (“susceptible, increased exposure”) and R for any of the included antibiotics [16,17], both S and I results are designated as “susceptible” for simplicity in this paper.

The Illumina WGS of all the strains was performed at the Brussels Interuniversity Genomics High Throughput core (www.brightcore.be), using the Nextera XT DNA Library Prep Kit (Illumina, San Diego, CA, USA) for library preparation. The raw Illumina reads were assembled using SPAdes [29].

Contigs shorter than 500 base pairs or with a coverage lower than 5 were filtered out, and the quality of the resulting fasta-files was assessed using Quast (v5.0.2) [30]. Assembled genomes were only included in the study if they fell within the quality criteria for WGS data of *Corynebacterium* spp., as outlined by Institut Pasteur’s BIGSdb (https://bigsdb.pasteur.fr/diphtheria/genomes-quality-criteria/, accessed on 14 January 2025).

DiphtOscan (v1.7.0) was then used to determine the presence of AMR genes, chromosomal mutations and integrons [31]. The --resistance_virulence, --extend_genotyping, and --integron options were used, with default minimum alignment identity (80) and coverage (50). The input genomes were screened for the presence of acquired resistance gene alleles and chromosomal mutations against two curated databases: the AMRFinder database (version 2024-07-22.1) and the DiphtOscan database specific to *C. diphtheriae* (version 2024-10-11.1) [32]. MLST were assigned using BIGSdb.

All genomes are publicly available on the BIGSdb Corynebacterium cgMLST database (see Supplementary Table S1 for IDs).

## 5. Conclusions

The accurate antibiotic susceptibility testing of *C. diphtheriae* is an important part of the control of diphtheria propagation (more so than the testing of *C. ulcerans*, which is less susceptible to spread among humans), while individual treatment of severe respiratory cases is mainly based on equine antitoxin administration. Antibiotics have more importance in the cure of cutaneous diphtheria, which is often multi-microbial [2].

Penicillins and macrolides remain the first-choice antibiotics for the treatment of diphtheria, and resistance is rare. However, antimicrobial susceptibility testing is needed for all toxigenic corynebacteria, especially *C. diphtheriae*, as resistance is emerging, but watchfulness should also be exercised for *C. ulcerans* as well. Alternative antibiotics should be tested as well.

## Figures and Tables

**Table 1:** Correlation of BMD categorical interpretation of MIC values and detection of AMR determinants.

Antimicrobial				
Agent	AMR Determinants ^1^	nP/nR ^2^	AMR Determinants ^1^	nP/nS ^3^
Benzylpenicillin	*pbp2m*	1/1	*blaOXA-2*	2/33
Amoxicillin	*pbp2m*	1/1	*blaOXA-2*	2/33
Cefotaxime	*pbp2m*	1/3	*blaOXA-2*	2/31
Meropenem	/		none	
Ciprofloxacin	*gyrA_S89F* + *qacEΔ*	6/10	*qacE*Δ, *qacL*	1/24
	*gyrA_S89F*	2/10		
	*qacEΔ*	2/10		
Erythromycin	*ermX*	3/3	none	
Clindamycin	*ermX*	3/3	none	
Tetracycline	*tet*(*33*)	9/13	none	
	*tet*(*33*) + *tet*(*W*)	2/13		
	*tet*(*65*)	1/13		
	*tet*(*Z*)	1/13		
Trimethoprim-sulfamethoxazole	*sul1*	17/29	*sul1*	1/5
	*sul1*, *dfrA1*	9/29		
	*sul1*, *dfrA16*	1/29		
	none	2/29		

^1^ AMR determinants: detected resistance genes or mutations. ^2^ nP/nR: number of strains with AMR determinants present/number of resistant strains. ^3^ nP/nS: number of strains with AMR determinants present/number of susceptible strains.

**Table 2:** *C. diphtheriae*: Percentage of resistant isolates on the basis of EUCAST interpretation.

Antibiotic	This Study (n = 34)	Marosevic et al. [10] (n = 298)	Berger et al. [16] (n = 200)
Benzylpenicillin	3	3	5
Amoxicillin	3	/	3
Cefotaxime (ceftriaxone *)	3	6	1
Meropenem	0	0	1
Erythromycin	9	4	5
Clindamycin	9	2	3
Ciprofloxacin	29	9	4
Tetracycline	38	/	38
Trimethoprim-sulfamethoxazole	85	/	33

* Results for ceftriaxone (Marosevic et al. [10] did not test cefotaxime).

**Table 3:** *C. ulcerans*: Percentage of resistant isolates on the basis of EUCAST interpretation.

Antibiotic	This Study (n = 33)	Marosevic et al. [10] (n = 123)	Berger et al. [16] (n = 200)
Benzylpenicillin	0	0	0
Amoxicillin	0	/	0
Cefotaxime (ceftriaxone *)	9	0	0
Meropenem	0	1	1
Erythromycin	0	2	1
Clindamycin	100	84	93
Ciprofloxacin	9	3	4
Tetracycline	0	/	3
Trimethoprim-sulfamethoxazole	0	/	0

* Results for ceftriaxone (Marosevic et al. [10] did not test cefotaxime).

## Data Availability

The whole genome sequences of all isolates are available on the DiphtOscan platform (see references of the sequences in Supplementary Table S1).

## Supplementary Materials

The following supporting information can be downloaded at https://www.mdpi.com/article/10.3390/antibiotics14020160/s1, Table S1: BIGSdb isolate ID of strain included in this study.
